# Batch Productions of Electrospun Janus Nanofibrous Films for Multifunctional Food-Packaging Applications

**DOI:** 10.3390/foods15193391

**Published:** 2026-09-23

**Authors:** Tingyu Chen, Qiuyun Guo, Hongyi Yin, Mengyao Wang, Hui Liu, Deng-Guang Yu

**Affiliations:** School of Materials and Chemistry, University of Shanghai for Science and Technology, 516 Jungong Road, Shanghai 200093, China; 243403094@st.usst.edu.cn (T.C.); 233393129@st.usst.edu.cn (Q.G.); 255393101@st.usst.edu.cn (H.Y.); 254203053@st.usst.edu.cn (M.W.)

**Keywords:** scale-up production, Janus structure, side-by-side electrospinning, freshness tracking, multifunctional membrane, intelligent food packaging

## Abstract

The development of intelligent packaging systems with dual monitoring and antibacterial capacities still poses one of the most substantial challenges for scientific advancement. In this study, polyvinylidene fluoride (PVDF) and grape skin extract (GSE) were employed as the carrier polymer and coloring agent in the round-side of electrospun Janus nanofibers, respectively, and titanium dioxide (TiO_2_) was used as the crescent-side active component. To overcome the bottleneck of low production efficiency in traditional needle-based electrospinning, an innovative “needles-in-holes” eccentric spinneret was developed, enabling side-by-side electrospinning for the first time and batch-scale fabrication of Janus-structured nanofibrous membranes with a 20-fold yield compared to traditional needle-based electrospinning. The resulting Janus membrane demonstrates high colorimetric sensitivity; superior hydrophobicity; excellent stability; and robust mechanical, antioxidant, and antibacterial properties. It exhibits a strong linear correlation in monitoring total volatile basic nitrogen and pH levels in river perch (*Perca fluviatilis*). Based on the pH-responsive properties of GSE, the Janus fiber membrane exhibited a distinct color change from off-white to light blue at 4 °C. Combined with the antibacterial effect of TiO_2_, the films effectively extended the shelf life of sea bass by 2 days. This work not only provides a high-performance multifunctional membrane for real-time freshness monitoring and preservation of fish products, but also demonstrates a batch-production strategy of side-by-side electrospinning, offering significant potential for intelligent food-packaging applications.

## 1. Introduction

Fish meat constitutes a vital source of nutrition, being rich in protein, fat, and minerals, and providing essential nutrients for humans [1,2]. High-value fish, such as sea bass, are rich in unsaturated fatty acids and have a high moisture content of over 70% [3], making them highly susceptible to spoilage during storage [4]. Perch spoilage is primarily attributed to three factors: rapid proliferation of psychrophilic bacteria [5,6,7], enzymatic activity within the fish, and lipid oxidation reactions. These processes result in the accumulation of spoilage markers [8], such as total volatile basic nitrogen (TVB-N) [9], trimethylamine (TMA) [10,11], and biogenic amines [12]. Traditional monitoring methods, such as high-performance liquid chromatography (HPLC) for biogenic amine detection or steam distillation followed by titration for TVB-N determination, are accurate but time-consuming and require specialized equipment [13]. These limitations restrict their application in real-time, non-destructive freshness monitoring within cold chain logistics [14,15]. Therefore, there is a pressing need to design smart packaging systems integrating indicator and preservation functions to boost quality-control efficiency for perch products [16,17,18].

Hydrophilic polymers are frequently utilized as pH-indicator carriers in food packaging [19], yet they are susceptible to water-induced swelling and structural deformation, which often results in dye leakage [20,21]. Furthermore, meat and seafood products are frequently stored under conditions of high humidity and low temperature, which may compromise the accuracy of freshness monitoring [22]. This limitation underscores the critical need for developing indicator systems based on hydrophobic polymer matrices [23]. Grape skin extract (GSE), extracted from natural sources, is safer and less harmful than synthetic chemical indicators [24]. GSE possesses a high anthocyanin content and is highly sensitive to pH changes, enabling it to serve as a colorimetric indicator for freshness monitoring [25]. Additionally, GSE contains phenolic compounds with antioxidant properties that may contribute to extending food shelf life [26,27]. Anatase-type titanium dioxide (TiO_2_) generates free radicals and oxidants upon light exposure [28,29]. These oxidants possess potent oxidizing properties that can disrupt bacterial cell membrane structures, thereby inhibiting bacterial growth and reproduction. The combination of GSE and TiO_2_ is hypothesized to result in a multifunctional composite, including enhanced antibacterial and antioxidant effects, and fresh monitoring.

Polyvinylidene fluoride (PVDF), known for its extreme hydrophobicity, also possesses favorable biocompatibility and outstanding mechanical characteristics [30,31,32]. However, its high hydrophobicity can lead to reduced reactivity. Electrospinning can effectively address this limitation [33,34]. It enables the functionalization of fiber membranes in a single step, allowing direct control over the morphology of the resulting products [35,36,37,38]. By inducing the stretching of polymer-solution jets via a high-voltage electrostatic field, electrospinning can efficiently prepare random fiber networks with diameters ranging from 50 nm to several micrometers and porosities exceeding 80% [39,40,41,42,43].

Based on the formats that the working fluids are guided into an electrical field, electrospinning can be categorized into two approaches: needle-based electrospinning and free-surface electrospinning [44]. The former excels at regulating complex multi-chamber nanostructures through the spinneret outlet, yet faces challenges in industrial-scale production. The latter facilitates large-scale manufacturing but struggles to produce intricate multi-chamber structures. Most recently, these two approaches were integrated into the third working fluid-guiding format, i.e., needles-in-holes multiple fluids’ simultaneous guiding format. The brand-new approaches have been demonstrated to be useful for creating homogeneous nanocomposites [45] and core–sheath nanofibers in a large-scale manner [46]. Thus, we hypothesized that the “needles-in-holes” spinneret for bulk production of Janus nanofibers could also be developed for combining the strengths of both traditional approaches.

The spinneret is the most critical component in an electrospinning equipment. In a sense, electrospinning techniques are named after their nozzles: for example, coaxial electrospinning based on the concentric spinnerets [47,48,49,50], side-by-side electrospinning based on eccentric spinnerets [51], and multi-fluid electrospinning based on multi-channel outlet spinnerets [52,53,54]. Although the core–sheath structure can achieve physical spatial separation, it typically involves the complete encapsulation of one component within another [55]. Positioning TiO_2_ on the outer layer would inhibit the color development of GSE, whereas placing GSE on the outer layer would render it susceptible to degradation. The parallel side-by-side arrangement of Janus structures effectively achieves physical spatial separation, preventing direct contact between the two components that could lead to inactivation, while ensuring the color development of GSE remains visible and the antibacterial function of TiO_2_ is preserved. This design ensures combined effects without mutual interference. Consequently, this study employs PVDF as the electrospun filament-forming matrix, with GSE incorporated into the round sides of the eccentric Janus nanofibers and anatase-type TiO_2_ incorporated into the crescent side. TiO_2_ exhibits favorable coverage and coloring properties; however, blending electrospinning of them may compromise the coloring ability of anthocyanins. The primary advantage of this design lies in its spatial isolation mechanism, which effectively mitigates the deactivation of indicators caused by redox reactions between anthocyanin phenolic hydroxyl groups and oxygen vacancies on the TiO_2_ surface [56,57]. When these components come into direct contact, anthocyanins undergo structural degradation via electron transfer, resulting in significant yellowing [58,59]. Different with the core–sheath and blended nanostructures, the Janus structure is uniquely able to make these components to remain independent and non-interfering while facilitating combined effects.

Within the above-mentioned contexts, this study aims to utilize side-by-side electrospinning to prepare multifunctional hydrophobic nanofibrous films in a batch manner based on a Patented spinneret. The nanofibrous films are expected to have the enhanced colorimetric, antioxidant, and antibacterial capabilities containing PVDF as a substrate, and GSE and TiO_2_ as the active ingredients. The resultant films are expected to be exploited as the smart active food packaging for simultaneously monitoring and preserving fish freshness. To overcome the limitations of traditional needle-based electrospinning in production efficiency and scalability, the innovative “needles-in-holes” integrated spinneret was exploited to develop the corresponding batch side-by-side electrospinning processes for the first time.

## 2. Materials and Methods

### 2.1. Materials

The river perch (*Perca fluviatilis*) was obtained from a market in Shanghai, China. PVDF pellets (M_w_ = 400,000 Da), ammonia water (NH_3_·H_2_O), sodium hydroxide (NaOH), hydrochloric acid (38%, AR), boric acid (≥99.5%), glycerol (≥99%, AR), and anhydrous potassium carbonate (≥99.0%, AR) were all procured from Maclin Biochemical Co., Ltd. (Shanghai, China). The grape skin extract (GSE) was purchased from Shanxi Haocheng Biotechnology Co., Ltd. (Taiyuan, China). Methyl red, methylene blue, *N*,*N*-dimethylformamide (DMF, AR, ≥99.5%), and acetone (AR, ≥99.5%) were obtained from Sinopharm Chemical Reagents Co., Ltd. (Shanghai, China). TiO_2_ nanoparticles (TiO_2_ NPs, 99.8% metal basis, 5–10 nm, anatase) were acquired from Aladdin Reagent Co., Ltd. (Shanghai, China). All experimental procedures employed deionized water.

### 2.2. Grape Skin Extract pH Sensitivity Test

The pH-sensitive solution was adjusted to the desired value using 0.1 mol L^−1^ hydrochloric acid and 0.1 mol L^−1^ sodium hydroxide. The absorbance was measured with a UV-visible spectrophotometer (Shimadzu UV2550, Kyoto, Japan) across wavelengths ranging from 400 to 700 nm.

### 2.3. Electrospinning

In this study, based on the traditional needle-based electrospinning, some pre-experiments were conducted to investigate the electrospinnability of working fluids, and the influences of varying anthocyanin concentrations (0%, 5%, 10%, and 15% in wt/wt of GSE to PVDF) on the resultant PVDF nanofibers (see Supplementary Information).

For the preparation of spinnable polymeric liquid, a ratio of 19% (*w*/*v*) PVDF solution was prepared by placing PVDF powder into a 3:2 (*v*:*v*) acetone/DMF solvent mixture as the stock solution. Subsequently, GSE powders and TiO_2_ nanoparticles could be added separately at different stock solutions to prepare another two kinds of working fluids. The working fluid containing TiO_2_ NPs was prepared by dispersing 1% (*w*/*v*) anatase-type titanium dioxide powders in the 19% (*w*/*v*) PVDF solution with continuous stirring. The nanosuspensions were subjected to ultrasonic treatment for over 30 min. These three kinds of fluids could be selected to organize various batch-production electrospinning processes based on the new concept-combined spinneret.

The homemade batch-production electrospinning apparatus comprised a high-voltage energy source (ZGF 60 kV/2 mA, Wuhan Huatian Co., Ltd., Wuhan, China), two syringe pumps (KSD100 and KSD200, Cole-Parmer, IL, USA), cardboard wrapped with an aluminum foil as the collector, and the new-concept “needles-in-holes” spinneret. All processes were performed under the ambient conditions of a temperature of 16 ± 2 °C and a relative humidity of 36 ± 5%.

During the batch-production processes, the round PVDF-GSE (PG) sides of the Janus nanofibers solidified from the working fluids, which were introduced into the electrical field through the stainless-steel needles. The crescent PVDF-TiO_2_ (PT) sides of the Janus nanofibers were co-solidified from the suspensions guided through the holes of the polypropylene tanks. The general injection rates of metal needles and holes were kept at 15 mL h^−1^ for the round sides and 10 mL h^−1^ for the crescents, respectively. The applied voltage was 30.0 kV. The fiber collection distance was fixed at 20 cm.

After about 2 h of batch electrospinning, a sheet of the Janus nanofibrous PG//PT membranes was fabricated. When the crescent-side solution was replaced with the TiO_2_-free solution, spinning was repeated under identical conditions for 2 h to obtain PG//P membranes. When the round-side solution was substituted with the GSE-free solution, and after 2 h of spinning under the same conditions, P//PT membranes were harvested. Finally, pure PVDF solutions lacking both GSE and TiO_2_ were used for the round and crescent sides, respectively, and after 2 h of spinning under the same conditions, P//P membranes were produced. The fiber membranes were dried in an oven at 40 °C for 24 h prior to subsequent testing.

### 2.4. Characterization

#### 2.4.1. Morphologies and Inner Structures

The morphological features of the Janus nanostructure were examined by scanning electron microscopy (SEM, JSM-IT500HR, JEOL, Akishima, Tokyo, Japan; FESEM, FEI Quanta 450, FEI, Hillsboro, OR, USA). Elemental composition of the surface fibers was determined by energy-dispersive spectroscopy (EDS, JEOL, Akishima, Tokyo, Japan). The internal architecture of the fibers was investigated using transmission electron microscopy (TEM, Thermo Talos F200X G2, Thermo Fisher Scientific, Waltham, MA, USA). The sample diameter was measured by analyzing 100 locations in the SEM images with ImageJ software V1.8.0 (National Institutes of Health, Bethesda, MD, USA).

#### 2.4.2. Fourier-Transform Infrared Spectroscopy

The Janus nanostructure underwent analysis via a Fourier-Transform Infrared Spectrometer (FTIR, PerkinElmer, Billerica, MA, USA). Spectra acquisition took place at a resolution of 2 cm^−1^, with a spectral range spanning from 500 cm^−1^ to 4000 cm^−1^.

#### 2.4.3. X-Ray Diffraction

X-ray diffraction (XRD) analysis was performed on the membranes with an X-ray diffractometer (D8 ADVANCE X, Bruker, Karlsruhe, Germany) operating at 40 kV and 40 mA. The scanning covered an angular range of 10° to 80°, proceeding at a rate of 5° min^−1^.

#### 2.4.4. Differential Scanning Calorimetry

Differential scanning calorimetry (DSC) was employed to investigate the thermal transition behavior of PVDF, GSE, and the composite fiber-membrane samples with different structures. During the measurements, the samples were subjected to heating and cooling scans under a nitrogen atmosphere, and the heat flow, as a function of temperature, was recorded. The melting temperature (Tm) was determined from the endothermic peak during the heating process, while the crystallization temperature (Tc) was determined from the exothermic peak during the cooling process.

The DSC curves were processed using Origin software 8.0. After baseline correction, the melting enthalpy (ΔH_m_) and crystallization enthalpy (ΔHc) were obtained by integrating the corresponding peak areas. The melting enthalpy represents the energy absorbed during the melting of the crystalline regions, while the crystallization enthalpy reflects the ability of the polymer chains to form crystalline structures during the cooling process. The relative crystallinity (X_c_) of the PVDF-based composite materials was calculated according to Equation (1):

(1) Xc = ΔH_m_/(ΔH_m_^0^ × W(PVDF)) × 100%

where Xc represents the relative crystallinity of PVDF (%), ΔH_m_ is the melting enthalpy of the sample (J/g), ΔH_m_^0^ is the theoretical melting enthalpy of fully crystalline PVDF (104.7 J/g), and W(PVDF) is the mass fraction of PVDF in the composite sample.

#### 2.4.5. Water Contact Angle

The water contact angle (WCA) of the membrane was measured using a contact-angle analyzer (JC2000C1, Shanghai Zhongchen digital technology equipment Co., Ltd., Shanghai, China). The images were then subjected to processing and analysis through ImageJ software to examine the dynamic behavior of water droplets on the fiber membrane.

#### 2.4.6. Water-Vapor Permeability

The water-vapor permeability (WVP) values of the nanofibrous films were assessed according to the ASTM E882-00 [60] method, with minor modifications. All samples were tightly wrapped around the nozzle of a 50 mL centrifuge tube and secured with a rubber band; each tube contained 25 mL of distilled water [61]. The tubes were maintained in an incubator set to 25 °C and 50% relative humidity. The tubes were weighed every 12 h for several days until the weight stabilized.

(2) $$\mathrm{WVP}=\frac{\Delta m\times x}{A\times \Delta P\times t}$$

In this formula, Δm represents the weight gain (g), x denotes the membrane thickness (m), *t* corresponds to the duration (h), A represents the opening area of the centrifuge tube (m^2^), and ΔP signifies the saturated water vapor-pressure difference between the centrifuge tube and the environment at the nanofibers (3179 Pa at 25 °C).

#### 2.4.7. Swelling Index and Water Solubility

The interaction between the fiber membrane and water molecules was evaluated by measuring the swelling index (SI) and water solubility (WS). Each dried sample (m_1_) was immersed in deionized water and stirred at 30 °C for 12 h [62]. After removing the water and wiping the surface (m_2_), the sample was oven-dried at 105 °C to a constant weight (m_3_). All measurements were conducted in triplicate, and the SI and WS values were calculated as follows:

(3) $$\mathrm{SI}\left(\%\right)=\frac{m_{2}-m_{1}}{m_{1}}\times 100$$

(4) $$\mathrm{WS}\left(\%\right)=\frac{m_{1}-m_{3}}{m_{1}}\times 100$$

#### 2.4.8. Tensile-Strength Test

Tensile strength (σ_u_) and elongation at break (εᵦ) were determined using a Precisionline vario tensile testing machine (Zwick Co., Ltd., Ulm, Germany). Prior to testing, fiber-membrane thickness was measured with a micrometer, and mechanical-property evaluation was conducted at a tensile speed of 1.0 mm min^−1^. Three independent tensile tests were performed for each membrane (n = 3). Ultimate tensile strength (σ_u_), elongation at break (ε_b_), Young’s modulus (E), and tensile toughness/fracture energy (U_t_) were extracted from the engineering stress–strain curves.

(1) Engineering stress and engineering strain. Engineering stress was calculated using the original cross-sectional area, while engineering strain was calculated relative to the initial gauge length:

(5) σ = F/A_0_

(6) ε = (L − L_0_)/L_0_ × 100%

where F is the tensile load, A_0_ is the original cross-sectional area of the specimen, L_0_ is the initial gauge length, and L is the gauge length during loading.

(2) Ultimate tensile strength (σ_u_). The ultimate tensile strength was taken as the maximum engineering stress on the stress–strain curve:

(7) σ_u_ = max(σ) = F_max_/A_0_

(3) Elongation at break (ε_b_). Because some curves contained residual sensor signals and low-stress tails after rupture, a consistent criterion was applied to all samples. The fracture strain was defined as the strain at which the post-peak stress first decreased to 20% of σ_u_ or below. For discrete data, the criterion can be written as follows:

(8) σ_i_ ≤ 0.2σ_u_ and σ_i−1_ > 0.2σ_u_ → ε_b_ = ε_i_

(4) Young’s modulus (E). A linear regression was performed over the initial stable linear region of each stress–strain curve, and Young’s modulus was taken as the slope of this region. Because strain was entered in Origin as a percentage (%), the fitted slope, k, has units of MPa/%. Therefore, Young’s modulus was calculated as follows:

(9) E = *d*σ/*d*ε = 100k

The fitting interval was selected according to the initial linear portion of each replicate, and a high R^2^ value was used as a quality check for the linear fit.

(5) Tensile toughness/fracture energy (*U*_t_). *U*_t_ was defined as the mechanical energy absorbed per unit volume before fracture, corresponding to the area under the stress–strain curve from 0 to ε_b_:

(10) *U*_t_ = ∫_0_^{ε_b_} σ dε

The area obtained directly from Origin has units of MPa% because strain is expressed as a percentage. Therefore, the integrated area was divided by 100 to convert percentage strain to dimensionless strain:

(11) *U*_t_ (MJ/m^3^) = Area (MPa·%)/100

#### 2.4.9. Gas Color Response of Fiber Membranes

The PG//PT membrane was positioned 1 cm above a solution containing 36% HCl and 28% NH_3_·H_2_O. This arrangement was maintained at 25 °C, and the color of the sensor tag was recorded using a colorimeter at 10, 20, 30, 40, 50, and 60 s intervals. The L, a, and b values of the tags were extracted from these membranes. The color-difference value (Δ*E*) was calculated from the extracted L (luminance), a (red/green), and b (yellow/blue) values.

#### 2.4.10. Fiber-Membrane Stability

The color stability of the fiber membrane was evaluated by comparing color changes between membranes stored in a refrigerator and those kept at room temperature. The composite membranes underwent a 14-day storage period in both refrigerated (4 ± 0.5 °C) and constant-temperature (25 ± 0.5 °C) environments. Refrigerated storage (4 °C): The membrane was kept in a refrigerator under complete darkness (no light exposure), as typical for cold chain storage of aquatic products. Ambient storage (25 °C): The membrane was stored under natural indoor lighting conditions in Shanghai (ambient daylight through windows; no direct sunlight). The samples were analyzed colorimetrically every two days to document the progression of color changes.

The Lab color space of composite membranes, defined by L, a, and b, was measured using a colorimeter (3nh SC-10, Shenzhen 3nh/ThreeNH Co. Ltd., Shenzhen, China). Total color difference (ΔE) was calculated using the following formula. Each sample underwent three measurements, with the average value taken to ensure statistical reliability:

(12) $$\Delta E=\sqrt{{\left(L^{*}-L\right)}^{2}+{\left(a^{*}-a\right)}^{2}+{\left(b^{*}-b\right)}^{2}}$$

where L*, a*, and b* are the colorimetric value parameters indicating the color of the membrane after a certain storage period. L, a, and b are the corresponding values of the pristine membrane.

#### 2.4.11. DPPH Antioxidant Activity

Following a previously reported DPPH method with slight adjustments, the membrane’s antioxidant capacity was evaluated [63]. Specifically, 20 mg of the membrane was soaked in 5 mL of methanol. Then, 1.5 mL of this extract was taken and added to 1 mL of a DPPH–methanol solution (25 mg L^−1^). After undergoing reaction at room temperature and under dark conditions for 30 min, the absorbance was measured at 517 nm. The percentage of DPPH radical scavenging activity was computed based on the following formula:

(13) $$\mathrm{DPPH}\;\mathrm{radical}\;\mathrm{scavenging}\;\mathrm{activity}\;(\%)\;=\;\frac{A_{0}-A_{1}}{A_{0}}$$

where A_0_ corresponds to the absorbance of the DPPH methanol solution, and A_1_ to that of the sample extract.

#### 2.4.12. Antibacterial Activity

The antibacterial performance of the membrane was assessed in vitro by examining its effects against Gram-negative *E. coli* and Gram-positive *S. aureus* using the inhibition-zone method. The samples were cut into circular discs with a diameter of 8 mm and placed into sterile disposable petri dishes according to their respective groups. The samples were then sterilized by ultraviolet (UV) irradiation for 60 min and stored for further use. *E. coli* and *S. aureus* suspensions were separately diluted to 10^6^ CFU/mL using sterile PBS solution, and 100 μL of each bacterial suspension was evenly spread onto LB agar plates. The sterilized samples were placed onto the surface of the culture medium and gently pressed with sterile forceps to ensure full contact between the samples and the medium. The plates were then incubated in a constant-temperature incubator at 37 °C for 24 h. After incubation, the plates were photographed with a standard camera, and the sizes of the inhibition zones were measured and recorded.

### 2.5. Application of Freshness Tracking for Perch

Place the PG//PT dual-function film next to the fish. The experiment was conducted under natural light conditions (indoor natural light coming through a window, avoiding direct sunlight) to simulate real-life retail or home storage environments. To assess the membrane’s impact on fish freshness, a test group was prepared by positioning a 1 × 1 cm^2^ membrane sample beside the perch. The test group and a blank control were then stored separately in a constant-temperature incubator (25 °C, 75% RH) and a refrigerator (4 °C). For the accelerated spoilage condition (25 °C), measurements were taken every half-day. For the refrigerated storage condition (4 °C), measurements were taken every two days (on days 0, 2, 4, 6, and 8) due to the slower spoilage rate. The freshness of the fish was evaluated by measuring TVB-N and pH levels according to Chinese standards GB 5009.288-2016 [64] (China) and GB 5009.237-2016 [65] (China), respectively. All measurements were performed in triplicate.

### 2.6. Statistical Analysis

All experiments were performed in three independent biological replicates (n = 3), with each replicate consisting of separately prepared samples. Data are expressed as the mean ± standard deviation (SD). We performed statistical analyses using analysis of variance (ANOVA). When appropriate, the Dunnett post hoc test was performed following ANOVA to compare treatment groups with the control. A *p*-value of less than 0.05 was considered statistically significant.

## 3. Results and Discussion

### 3.1. Batch Production of Janus Nanofibers by Side-by-Side Electrospinning

Traditionally, the side-by-side spinneret consists of two parallel metal capillaries [66,67]. When two parallel working fluids are pumped out from the spinneret, the two working fluids inevitably repulse each other due to the fact that they have the same electrical properties. Thus, the creation of integrated Janus nanofibers once posed one of the most difficult challenges. This case resulted in extremely limited Janus nanofibers-based functional nanomaterials, showing a sharp contrast with abundant reports about coaxial electrospinning and electrospun core–sheath nanofibers [68,69]. Yu et al. [70] resolved this issue using an eccentric spinneret with a Chinese authorized patent, by which the electrical repulsion can be effectively prevented for forming an integrated side-by-side Taylor cone and, later, the integrated Janus nanofibers. Most recently, a new-concept third type of electrospinning was developed, in which the traditional needle-based electrospinning and free-surface needless electrospinning can be integrated for large scale productions of complex nanostructures and nanodevices [44,45,46]. Batch production of complex nanostructures is one of the most difficult and important challenges for nanofabrication. Based on this new concept, and also along the previous eccentric patent, for the first time, a brand-new batch-production method for creating Janus nanofibers was patented by Yu et al. [71]. Here, we pioneered its potential application in creating smart food-packaging films.

Based on the eccentric needle-based spinneret, here, a new-concept “needles-in-holes” batch-produced spinneret was developed for implementing the large-scale side-by-side electrospinning processes. A diagram about the integrated spinneret is shown in Figure 1a. The more reasonable arrangements of the two capillaries in the eccentric nozzle than the traditional parallel nozzle are included in the upper-right inset. Several digital photographs about the spinneret are shown in Figure 1b–f. The main body consists of a horizontally positioned cylindrical polypropylene tank containing multiple parallel stainless-steel capillaries with an inner diameter of 0.6 mm. This study demonstrates a four-channel side-by-side spinneret, where capillaries are uniformly distributed within the tank, with a center-to-center spacing of about 3.0 cm. This arrangement ensures the stability and uniformity of multiple jet distributions within the electric field. All capillaries converge at a common fluid inlet at the top of the tank. Their bottoms protrude uniformly about 1.0 mm beyond the tank’s lower surface, forming parallel fluid outlets of consistent diameters. Corresponding holes are drilled in the tank bottom at matching positions. This kind of structural spinneret facilitates the smooth entry of both the round (the metal needles) and the crescent-shaped sides (the holes under the polypropylene tank) working fluids into the electric field region.

During the electrospinning process, specific safety hazards exist. The entire nozzle assembly is sealed and fixed with epoxy resin, ensuring structural stability and electrical insulation. As diagrammed in the upper-right inset of Figure 1d, among all the utilized metal capillary needles, only a small section of one metal capillary was exposed for introducing the high voltage to the working fluids, while all other sections were completely covered using epoxy resin (the adhesive) for an effective electrical insulation. This exposed charged metal becomes a strong corona discharge point. Provided there are two adjacent points for discharging, it is highly prone to generating electrical sparks. Simultaneously, volatile organic solvents (e.g., acetone and ethanol) within the polymer solution ejected from the needle rapidly vaporize, forming localized high-concentration flammable vapor zones around the nozzle. When electrical sparks contact these vapors, they can trigger instantaneous localized combustion or open flames (Figure S1). This phenomenon not only severely disrupts the stability of the Taylor cone and the continuity of the jet, leading to uneven fiber formation, but also poses potential fire and explosion hazards. This underscores the critical importance of equipment insulation and safety design.

Figure 1b shows a cylindrical storage tank with four bottom holes for the crescent-shaped side spinnable suspensions, fabricated by bonding two 20 mL syringes with epoxy resin. Figure 1c displays a photograph of the complete “needles-in-holes” dual-fluid parallel configuration controlling the spinneret, with the syringe connected via a highly elastic silicone tube. Figure 1d presents an overall physical diagram of the self-made large-scale production “needles-in-holes” spinneret, featuring a compact and lightweight structure for easy installation and operation. Theoretically, the electrospinning process should fully utilize all input electrical energy to achieve solution stretching and fiber solidification; however, in practice, some energy inevitably dissipates into the environment. Traditional metal spinnerets often suffer from energy-dissipation issues. This new-concept spinneret significantly improves energy-utilization efficiency by combining a rational structure with non-metallic materials and applying an epoxy-resin coating to its surface. Figure 1e illustrates a round-side working fluid-inlet structure composed of four fused metal capillaries enclosed within an outer plastic shell, through which the round-side solution is uniformly dispersed into the four metal capillaries. Figure 1f depicts the four parallel orifices at the tank bottom, comprising four eccentrically offset metal capillaries positioned within corresponding holes. Crescent-shaped suspensions enter the horizontally positioned tank from one side, collectively forming a dual-fluid parallel flow pattern that directs both streams into a high-voltage electrostatic field. Figure 1f also present the enlarged views of four outlets.

A diagram about the key elements in the homemade apparatus is shown in Figure 1g, which contains mainly the spinneret, two syringe pumps, a collector, and a power supply. Some typical details about the working processes are illustrated in Figure 1h–l. Figure 1h depicts the side-by-side electrospinning apparatus used in this study, which was implemented for the preparations of PVDF-GSE//PVDF-TiO_2_ (PG//PT) Janus nanofibers. The crescent-shaped-side PVDF-TiO_2_ suspension was driven by the first syringe pump and injected directly into a horizontally positioned reservoir via silicon tubing; this reservoir functions as a fluid distribution chamber, evenly distributing the spinning solution to four outlets at its bottom. The round-side solution, driven by the second syringe pump, was directed through another silicon tube into four separate stainless-steel capillary tubes. At each outlet, the round-side solution emerged from the capillary tips, while the crescent-side solution flowed from the reservoir holes. The two solutions came into close parallel contact at the outlet, forming an initial two-fluid droplet. For safety, all components must be grounded to prevent possible electric shock. The spinneret was hung on a metal stick covered using Teflon, with the high-voltage power source clamped to the exterior of the metal capillary via alligator clips, enabling efficient transfer of electrostatic energy to the working fluids (as shown in Figure 1i).

For comparisons, four kinds of Janus nanofibers with various encapsulations of GSE and TiO_2_ in both sides were fabricated, i.e., PVDF-PVDF (P//P), PVDF-GSE//PVDF (PG//P), PVDF//PVDF-TiO_2_ (P//PT), and PG//PT. With the fabrications of PG//PT nanofibers as an example, the batch side-by-side electrospinning processes are shown in Figure 1h–l. These digital photos illustrate three characteristic stages following fluid ejection: Taylor cone formation, straight fluid jet, and instable bending and whipping region. Magnified images of the Taylor cone reveal that, under the applied voltage of 30 kV, each outlet has a stable compound Taylor cone there. Its round side is rich in anthocyanins and appears brown, while the other crescent side is white because it contains TiO_2_ (Figure 1j,k1–k4). Under the electric field, the jets are rapidly stretched, fragmented, and dried, ultimately solidifying into continuous nanofibers (Figure 1(l1–l4)). This multi-hole-design spinneret enables the simultaneous production of Janus nanofibers from multiple nozzles in a single step and in a straightforward manner, significantly enhancing the preparation efficiency and structural uniformity of nanofibrous membranes, providing a feasible technical pathway for industrial-scale continuous production. The dynamic electrospinning process based on the mass-produced “needles-in-holes” spinneret is detailed in Supplementary Video S1.

Integrated circuits offer a range of advantages over conventional discrete circuits, namely miniaturization, high integration density, small size, high reliability, low power consumption, low cost, and high performance. Similarly, the present integrated electrospinning process is expected to offer many advantages for the batch production of complex nanofibers. Here, the general flow rate of double fluids was 25 mL/h. Compared with traditional needle-based electrospinning, where the flow rate is only about 1.0 mL/h [33], the yield was increased more than 20-fold. In future, a hundred- or even thousand-fold increase in yield may be readily achieved by increasing the number of holes in the integrated spinneret, without the need for additional pumps or fluid drivers.

### 3.2. Outer Morphologies and Inner Nanostructures

The efficacy of GSE solution as a pH indicator was validated by examining its color-changing properties across different pH values and its corresponding ultraviolet–visible absorption spectra, as shown in Figure S2. We investigated the effect of anthocyanin concentration on PVDF electrospinning, as shown in Figure S3. When the anthocyanin-to-PVDF concentration ratio was 1:10 (wt/wt), the spun fibers exhibited the highest uniformity. Subsequently, an anthocyanin concentration of 10% was adopted as the optimal formulation.

Figure 2a–d show SEM images of Janus P//P, PG//P, P//PT, and PG//PT nanofibers prepared via side-by-side electrospinning. Figure 2b shows that anthocyanin incorporation induces a smooth PVDF fiber surface morphology. In the P//PT system (Figure 2c), TiO_2_ nanoparticles are anchored and homogeneously distributed on crescent PVDF sides through ultrasonic dispersion and the high-voltage field in electrospinning. Figure 2d–k are characterizations about PG//PT nanofibers. These Janus nanofibers have a homogeneous elemental distribution of C, F, O, and Ti, respectively (Figure 2(e1–e5)), and an average diameter of 0.66 ± 0.16 μm (Figure 2f). Notably, direct contact between TiO_2_ and anthocyanin causes yellowing, yet side-by-side electrospinning constructs an isolation structure, preventing their contacts. EDS analysis of PG//PT fibers (Figure 2g,h) also confirms the four elements in the Janus structure. Prominent fluorine (40.8%) and carbon (52.3%) peaks correspond to the PVDF matrix, while the increased carbon/fluorine ratio (1.28) confirms anthocyanin presence. The detected titanium peak (1.9%) confirms the existence of TiO_2_.

Figure 2i presents the TEM image of the Janus fiber, exhibiting a marked contrast in gray levels across the green line. Figure 2j is an enlarged image from Figure 2i, which provides a clearer view of the two side-by-side chambers, with one side containing the TiO_2_ nanoparticles. The difference of gray level stems from the material disparity between the round and crescent regions, affirming the successful creation of the eccentric Janus fiber with evident particles on its outer surface. A diagram showing the four TiO_2_ nanoparticles is drawn in Figure 2k. The above findings confirm that the Janus structure has effectively achieved spatial separation between anthocyanins and TiO_2_. Additionally, to verify the uniformity of the Janus nanofibrous membranes, we performed SEM imaging of four batch-spun Janus fiber membranes at different extrusion positions (Figure S4) and conducted statistical analysis of the fiber diameters. The results revealed consistent fiber morphology and diameter across different sampling regions, with no significant agglomeration or structural defects observed. This demonstrates that the parallel spinning process exhibits excellent reproducibility and structural uniformity, making it suitable for large-scale production.

### 3.3. Physicochemical Characterization of Nanofibrous Membranes

The XRD patterns in Figure 3a show that all samples exhibit characteristic peaks at 2θ ≈ 18.6°, 20.1°, and 26.8°, corresponding to the (020), (110), and (021) crystal planes; notably, the PVDF characteristic peaks in the GSE-containing samples exhibit a slight rightward shift, indicating that GSE molecules were successfully incorporated into the PVDF matrix and may have induced minor lattice distortions in the PVDF crystal structure through intermolecular interactions. Additionally, the sample containing anatase-type TiO_2_ exhibits a distinct characteristic peak corresponding to the anatase TiO_2_ (101) crystal plane at 2θ ≈ 25.3°, confirming its successful loading. The infrared spectrum (Figure 3b) further indicates characteristic vibration peaks of the piezoelectric active β-phase of PVDF at 837, 880, and 1279 cm^−1^, corresponding to CF_2_ bending, CH_2_ rocking, and asymmetric CF_2_ stretching, respectively. Peaks at 760, 795, 975, 1072, 1174, and 1402 cm^−1^, respectively, correspond to the characteristic peaks of the PVDF α-phase; additionally, the Ti-O-Ti stretching vibration peak appearing at ~760 cm^−1^ further confirms the presence of anatase TiO_2_. The above results demonstrate that electrospinning successfully prepared composite fibers containing a PVDF matrix, a GSE functional round side, and a TiO_2_ crescent side, with the key components GSE, anatase TiO_2_, and PVDF crystal phases (α and β) effectively introduced and retained.

Figure 3c shows the heating DSC curves of PVDF, GSE, and the various Janus nanofibrous films. In the heating process, all samples exhibited distinct endothermic melting peaks, indicating the presence of crystalline structures in the prepared samples. The PVDF sample showed typical melting behavior of a semi-crystalline polymer, with a clear melting peak located at approximately 167 °C. Compared with PVDF, the melting peak of GSE shifted toward a lower temperature, indicating that GSE possesses different thermal transition characteristics from PVDF. The P//P Janus nanofibers exhibited a similar melting behavior to PVDF, suggesting that the electrospinning process did not significantly disrupt the original crystalline structure of PVDF. The PG//P, P//PT, and PG//PT Janus nanofibers similarly showed obvious melting endothermic peaks. However, slight differences in peak position and peak area were observed among these samples, indicating that the interfacial interactions between different components and the fiber structures influenced polymer chain arrangements and crystal-growth behaviors.

Water contact angle (WCA) measurement is a critical metric for quantifying surface wettability and assessing the affinity of solids for water. All membranes exhibit WCA values exceeding 120°, due to the ultra-low surface energy of the –CF_2_ groups in PVDF. P//P, PG//P, P//PT, and PG//PT Janus nanofibrous films had a WCA value of 134.97 ± 1.75°, 122.40 ± 0.98°, 124.30 ± 1.45°, and 120.57 ± 0.97°, respectively (Table 1). The abundant hydroxyl moieties of anthocyanin significantly reduce the contact angle, attenuating hydrophobicity, whereas anatase TiO_2_, bearing surface hydroxyl groups that strongly hydrogen-bond with water, further promotes hydrophilic character. Consequently, membranes incorporating anthocyanins and TiO_2_ exhibit diminished yet still pronounced hydrophobicity.

Water-vapor permeability (WVP) quantifies the quantity of water vapor that a material permits to pass through per unit time, used to assess its barrier properties or breathability; a lower WVP indicates higher moisture resistance. The WVP test results are shown in Figure 3d and Table 1. The C–F bonds in PVDF have extremely high bond energy and low polarizability, forming a dense electronic cloud barrier that effectively blocks water molecule penetration, resulting in a low WVP value 0.47 ± 0.01 (10^−7^ g m^−1^ h^−1^ Pa^−1^). The introduction of PG//P, rich in hydrophilic hydroxyl anthocyanins, allows GSE molecules to adsorb water molecules via hydrogen bonding, forming local hydrophilic channels. Simultaneously, moisture absorption and swelling cause fiber pore expansion, resulting in a WVP value of 0.88 ± 0.02 (10^−7^ g m^−1^ h^−1^ Pa^−1^). The introduction of TiO_2_ in P//PT fiber membranes also increases water-vapor permeability, inducing interface defects that disrupt structural density in the PVDF matrix, while surface Ti–OH groups form polar adsorption channels, collectively leading to a WVP value of 0.78 ± 0.02 (10^−7^ g m^−1^ h^−1^ Pa^−1^). Notably, although the WVP value of the Janus-structured PG//PT membrane reaches the highest level among the three, it remains significantly superior to common food-packaging materials, indicating its excellent water vapor-barrier capability [72].

Figure 3e and Table 1 reveal that the swelling index (SI) and water solubility index (WS) of the various fiber membranes exhibit pronounced variations. Because meat products are typically stored under high humidity and low-temperature conditions, evaluating the structural integrity and water stability of the membrane is essential. Lower SI and WS values signify superior structural integrity and water stability. Pure PVDF displays optimal stability, attributable to the combined resistance of its intrinsic hydrophobicity and high *β*-phase crystallinity toward water penetration, yielding an SI of 4.67 ± 2.08%. Consequently, WS approaches zero, owing to the combined influence of inert C–F bonds and the superhydrophobic surface. Upon GSE incorporation, the SI of PG//P membranes rose to 76.90 ± 5.00%. This increase results from the pronounced hygroscopicity of the hydrophilic hydroxyl moieties of GSE, generating swelling stress that compresses the sheath layer. WS concomitantly increased as the PVDF sheath failed to fully encapsulate the hydrophilic GSE domains, thereby precluding their complete isolation.

For TiO_2_-containing systems, the P//PT membrane exhibited an SI of 12.93 ± 1.31%, surpassing that of pristine PVDF yet markedly inferior to PG//P. Rigid TiO_2_ nanoparticles constrain polymer-chain mobility; nevertheless, their inherent hydrophilicity and interfacial defects promote localized swelling. Importantly, the Janus PG//PT architecture lowers both SI and WS (29.1 ± 3.15% and 1.18 ± 0.15%, respectively) relative to PG//P, attributable to the physical confinement imparted by TiO_2_ within the crescent side. Despite the potential of TiO_2_ hydrophilicity to facilitate water adsorption, the densely packed nanoparticles create a tortuous barrier within the crescent layer that markedly extends the diffusion pathway. Meanwhile, the reduced swelling index and water solubility of the PG//PT membrane compared to PG//P may indicate that the TiO_2_-containing crescent side physically constrains the polymer matrix, which could help retain functional components within the fiber structure.

Figure 3f and Table 1 present the stress–strain profiles of the P//P, PG//P, P//PT, and PG//PT Janus nanofibrous films. The results in the Table 1 are reported as mean ± SD. Figure 3f shows one representative stress–strain curve for each group to facilitate comparison of the overall mechanical responses of the different membranes. The data reveal that anthocyanin molecules readily infiltrate and occupy the nanoscale free volume and inter-chain pores of the PVDF matrix [73]. This infiltration mitigates structural defects and thereby increases matrix density. Anthocyanins possess abundant polar groups—namely hydroxyl (–OH) and carbonyl (C=O) groups—while PVDF chains carry highly electronegative fluorine atoms (–F). These moieties establish strong hydrogen bonds (C=O···H–O, C–F···H–O) and dipole–dipole interactions with the fluorine atoms of PVDF. Well-dispersed anthocyanin molecules act as physical cross-linkers within the amorphous domains of PVDF. Their presence increases the local chain density, thereby enhancing intermolecular entanglement. Quantitatively, P//P fibrous films exhibited the highest elongation at break (90.14 ± 20.58%), indicating relatively high chain mobility and plastic deformability in neat PVDF. However, its ultimate tensile strength (0.789 ± 0.296 MPa) and Young’s modulus (2.80 ± 2.28 MPa) were comparatively low. After introducing the GSE-related side in P//PG nanostructures, σ_u_ increased to 1.510 ± 1.217 MPa, E increased to 4.77 ± 2.25 MPa, and Ut increased to 0.621 ± 0.681 MJ/m^3^. Compared with P//P, PG//P therefore exhibited higher strength and stiffness, while its elongation at break decreased to 52.87 ± 34.51%. This behavior suggests a transition from a highly extensible, low-load-bearing response toward a stronger and stiffer structure after GSE incorporation.

However, adding TiO_2_ caused a reduction in the mechanical properties of the composite fibers. Although TiO_2_ nanoparticles are uniformly dispersed on the fiber surface, their role as rigid inorganic fillers may lead to insufficient interface compatibility with the flexible PVDF polymer matrix. These weak interface regions can become stress-concentration points under loading, triggering the initiation and propagation of microcracks, thereby degrading the overall mechanical properties of the material. Additionally, the presence of TiO_2_ particles may partially hinder the slippage and rearrangement of PVDF molecular chains and limit the plastic deformation capability of the material. In contrast, P//PT nanofibrous films showed a clear decrease relative to F3 PG//P: σ_u_ decreased from 1.510 to 1.030 MPa, E decreased from 4.77 to 1.06 MPa, and U_t_ decreased from 0.621 to 0.287 MJ/m^3^, while ε_b_ changed only slightly. This trend is consistent with the interpretation that TiO_2_ nanoparticles can disturb PVDF chain entanglement and weaken effective load transfer when introduced into a GSE-containing system.

PG//PT showed a comparable mechanical performance with PG//P Janus fibrous films, having a σ_u_ value of 1.692 ± 0.188 MPa, an E value of 3.96 ± 1.09 MPa, and a U_t_ value of 0.524 ± 0.175 MJ/m^3^. Although TiO_2_-containing sides may reduce macroscopic strength, the GSE-PVDF sides could effectively compensate the reductions. Overall, the four Janus films suggest that GSE is beneficial for reinforcing the PVDF matrix, whereas TiO_2_ nanoparticles may disturb PVDF chain entanglements and the continuity of load-transfer pathways to weaken the mechanical performances.

### 3.4. Color Development and Freshness-Preservation Capabilities

Before using it for monitoring meat freshness, the color-indicator reaction of volatile ammonia is an important criterion. As shown in Figure 4a, under 38% HCl conditions, the fiber membrane changes from off-white to pink. Under 28% NH_3_·H_2_O conditions, the PG//PT membrane causes the fiber membrane to change from beige to yellow; the released ammonia combines with water vapor and enters the fiber layer, forming hydrated ammonia, which leads to changes in the structure and color of anthocyanins (Figure 4b).

The color stability of the fiber membrane is a key indicator ensuring the reliability and practicality of food freshness-monitoring results, directly affecting the accuracy, repeatability, and long-term applicability of the detection system. As is well known, anthocyanins, as natural pH-responsive pigments, are susceptible to environmental factors such as light intensity, temperature fluctuations, pH changes, and oxidative effects, which can affect their molecular structural stability. Therefore, the variation in ΔE of the membrane at 4 °C and 25 °C was studied to evaluate the stability of anthocyanins in food-freshness monitoring. Generally, when ΔE > 5, it is considered a noticeable color difference to humans. The membrane was stored under two distinct conditions: (i) refrigerated storage at 4 °C in complete darkness (typical for cold chain logistics), and (ii) ambient storage at 25 °C under natural indoor lighting in Shanghai (ambient daylight through windows without direct sunlight.). Color changes were measured bi-daily throughout 14 days. As shown in Figure 4c, the fiber membrane exhibited negligible visible color change after 14 days under both storage conditions, indicating excellent visual stability. The corresponding quantitative color difference values (ΔE) are presented in Figure 4c; the maximum ΔE change was 3.78, far below the visual recognition threshold. The observed color stability of the PG//PT membrane over 14 days of storage may suggest that the hydrophobic PVDF matrix and the Janus configuration help protect anthocyanins from environmental degradation.

Polyphenolic compounds are one of the primary contributors to antioxidant capacity [74]. DPPH is a synthetic stable radical commonly used to assess the antioxidant capacity of substances. When antioxidants provide hydrogen atoms or electrons, DPPH is reduced to colorless DPPH-H, and the degree of solution decolorization is positively correlated with antioxidant capacity. The antioxidant properties of the membrane are shown in Figure 4d. The increase in GSE improved the antioxidant performance of the membrane to 75.345%; TiO_2_ also exhibited notable antioxidant performance, but it was lower than that of GSE.

The antibacterial activity of electrospun fiber membranes against *Escherichia coli* (*E. coli*) and *Staphylococcus aureus* (*S. aureus*) was determined using the inhibition-zone method. The results are included in Figure 4e–g. Both GSE and TiO_2_ exhibited antibacterial activity against *S. aureus* and *E. coli*. TiO_2_ demonstrated stronger broad-spectrum antibacterial activity compared to GSE. This is because TiO_2_ inherently possesses antibacterial activity against both Gram-positive and Gram-negative bacteria and fungi. Additionally, the fibrous PG//PT films loaded with both GSE and TiO_2_ exhibited a significant enhanced antibacterial effect, with an average zone of inhibition (ZOI) diameter of 25.0 ± 1.0 mm for *S. aureus* (Figure 4f) and 18.7 ± 1.2 mm for *E. coli* (Figure 4g). The superior antibacterial activity of the PG//PT membrane may result from the complementary effects of both GSE and TiO_2_. The incorporated TiO_2_ nanoparticles might contribute to bacterial inhibition through surface contact and physical interaction with bacterial cells, while any additional contribution from photoinduced reactive oxygen species generation would depend on light conditions, which were not quantitatively controlled in this study. The exact mechanisms underlying the observed antibacterial effect warrant further investigation.

Additionally, the Janus-structured packaging system may exhibit environmentally dependent antibacterial regulation based on ambient-light conditions. Figure 5 illustrates a proposed combined antibacterial mechanism of PG//PT fiber membranes against bacteria, along with their smart monitoring and preservation functions in food packaging. In illuminated settings such as retail displays, the system could leverage a photocatalytic mechanism wherein UV-activated anatase TiO_2_ generates reactive oxygen species (ROS), potentially oxidizing and compromising bacterial cellular integrity [75]. Concurrently, GSE might contribute not only by disrupting microbial membrane integrity (via its phenolic hydroxyl groups and specific inhibition of teichoic acid biosynthesis in Gram-positive bacteria) but also possibly act as a photosensitizer, broadening light absorption and augmenting the photonic quantum efficiency of TiO_2_, thereby intensifying the overall photocatalytic antibacterial performance. Under low-light or dark conditions, such as during cold chain transit, GSE would likely assume a dominant role through non-photoinduced antibacterial actions: its molecules may integrate into bacterial biomembranes via hydrogen bonding and hydrophobic interactions, persistently inhibiting metabolic enzymes and disrupting cell wall formation. Although the photocatalytic function of TiO_2_ likely diminishes under dark conditions, its nanoparticles may continue to suppress microbial activity through surface adsorption and physical contact, thereby helping to maintain the antibacterial effect of GSE. This spatiotemporally coordinated operation, enabled by the Janus architecture, is expected to ensure consistent and high antibacterial performance across varying light conditions. In addition to potentially controlling spoilage microorganisms in aquatic products such as perch, the system also offers real-time visual freshness monitoring via its pH-responsive color-changing property, underscoring its significant potential in smart food packaging and preservation.

### 3.5. Fish Meat Freshness Tracking Application

Place the PG//PT multifunctional fiber membrane side by side with the perch fillets. By evaluating the visible appearance of the fish meat and the color transformations of the indicator membrane between the blank control group and the membrane-treated group at 25 °C and 4 °C, this study explores the dual roles of the membrane in perch preservation and freshness signaling. As shown in Figure 6a, the fish meat in the blank control group changed from a bright translucent color to a deep yellow color, with noticeable mucus formation beginning on day 1.5, and the meat color turning yellow. In contrast, the membrane group exhibited lower levels of fish meat spoilage compared to the blank control group, with no noticeable mucus formation within 2 days and a pale-yellow color. As the fish meat deteriorated, the indicator membrane changed from beige to blue-green. This change showed a good correlation with the degree of fish meat spoilage. Additionally, in an 8-day experiment conducted at 4 °C, as shown in Figure 6b, the fish meat in the blank control group spoiled on the 6th day, while the fish meat in the membrane group did not show obvious signs of spoilage on the 6th day, and the membrane color changed from beige to light blue. This further confirmed its freshness-indicator function.

Figure 6c shows the changes in TVB-N (volatile basic nitrogen), pH, and color-difference value (ΔE) of perch over storage time at 25 °C and 4 °C. Microbial metabolism and endogenous enzymatic degradation lead to protein breakdown, generating TVB-N and increasing pH. Therefore, TVB-N and pH serve as core chemical indicators for evaluating food freshness, with their dynamic changes accurately reflecting the degree of fish spoilage. According to the classification criteria reported in the literature [16], the freshness of perch can be divided into two levels based on TVB-N values: fresh (≤15 × 10^−2^ mg g^−1^) and sub-fresh (15–20 × 10^−2^ mg g^−1^). TVB-N values exceeding 20 × 10^−2^ mg g^−1^ indicate spoilage. This grading system is significantly correlated with the sensory quality of fish meat and microbial-growth conditions. The TVB-N values and pH of fish meat undergo significant changes at 25 °C and 4 °C. At 25 °C, the TVB-N value on day 0 was 5.84 ± 0.68 × 10^−2^ mg g^−1^, indicating that the fish meat was fresh; after 1 day, the TVB-N value was 19.53 ± 0.80 × 10^−2^ mg g^−1^, indicating that the fish meat was gradually deteriorating, with the membrane color changing from white to light blue (ΔE = 5.83 ± 0.07). After 2 days, the TVB-N value was 34.93 ± 0.56 × 10^−2^ mg g^−1^, indicating that the fish meat had deteriorated, and the membrane color changed from light blue to blue-green (ΔE = 9.00 ± 0.06); this result indicates that, at room temperature (25 °C), the indicator fiber membrane can visually reflect the spoilage process of fish meat through color changes. At 4 °C, the TVB-N content of the blank control group on day 6 was 25.39 ± 0.43 × 10^−2^ mg g^−1^, indicating that the fish meat had spoiled, while the TVB-N content of the membrane-applied group did not spoil until day 8 (ΔE = 8.07 ± 0.19). This result suggests that, at 4 °C, the indicator membrane may help delay fish meat spoilage and potentially extend the shelf life by approximately 2 days, based on the chemical spoilage indicators TVB-N and pH. Therefore, this indicator membrane has dual functions in preserving the freshness of perch and indicating its freshness. It should be noted that the two-day extension of shelf life was attributed to the overall effect of the PG//PT membrane, including the contributions of its individual components, GSE and TiO_2_, and of the electrospun Janus configuration.

The correlation between the color difference (ΔE) of the PG//PT membrane and the fish TVB-N content/pH value is presented in Figure 6d, demonstrating its utility in effectively assessing the membrane’s ability to indicate freshness. A clear linear positive correlation is observed between ΔE and both freshness indicators. The regression equation for the TVB-N/ΔE group is y = 0.30725x − 0.60752, with a correlation coefficient of R^2^ = 0.93818; the regression equation for the pH/ΔE group is y = 5.21242x − 28.97198, with R^2^ = 0.94142. Based on this, the color state of the PG//PT membrane can be used to reliably monitor changes in the freshness of perch during storage. By the way, the calibration between ΔE and TVB-N/pH was established specifically for river perch under the investigated storage conditions, and further validation would be required before its application to other fish species, storage temperatures, or packaging conditions.

In recent years, the field of food packaging has been rapidly advancing toward active packaging systems with intelligent and multifunctional properties. These continuous developments require, on the one hand, novel raw materials for such applications—including organic salts, sodium lactate, buriti (*Mauritia flexuosa* L.) oil, and starch [76,77,78,79]. On the other hand, advanced techniques are continuously being introduced into this promising scientific field that are expected to be green, safe, and sustainable. Nanofabrication based on biopolymers for various applications has become one of the most critical sustainability concerns over the past several decades [80,81,82]. The present protocols offer an alternative route for the sustainable development of potential smart food packaging with multiple functional performances. By the way, PVDF was exploited as the polymeric matrix due to its fine filament-forming property, its super hydrophobicity and stability for supporting the color reaction of GSE, and the good process–structure–performance relationship [83,84]. However, PVDF has the negative concerns associated with environment contaminant [85,86] and safety of F migration [87,88], which are also faced by its many other applications. Therefore, it should be noted that the present study does not include specific migration experiments for TiO_2_ nanoparticles, fluorinated compounds, or GSE components. The potential release of these substances from the PG//PT membrane and their transfer to food simulants or actual food products may require systematic evaluation according to established standards (e.g., EU 10/2011 or FDA regulations) before any future application in direct food contact.

## 4. Conclusions

In this study, a Janus-structured PG//PT composite fiber membrane was successfully fabricated via side-by-side electrospinning, integrating pH-sensitive grape skin extract (GSE) as a colorimetric indicator and antibacterial TiO_2_ as a preservative agent. The hydrophobic PVDF matrix and the spatial segregation of GSE and TiO_2_ within the Janus architecture effectively prevented chromatic interference and enhanced functional stability. The resulting membrane exhibited excellent hydrophobicity, low water-vapor permeability, suppressed swelling, and strong mechanical properties. It enabled real-time visual monitoring of perch freshness, with strong linear correlations between color difference (ΔE) and both TVB-N and pH (R^2^ = 0.938 and 0.941, respectively), clearly distinguishing three freshness stages. Moreover, the membrane exhibited an enhanced antibacterial effect against *S. aureus* and *E. coli*, remarkably extending the shelf life of river perch (*Perca fluviatilis*) by approximately two days at 4 °C. Importantly, this work may help address two key challenges—anthocyanin leaching and TiO_2_–induced discoloration—through the combined use of a hydrophobic matrix and Janus configuration. Furthermore, a “needles-in-holes” combined spinneret was adopted for batch-scale production of Janus nanofibers that may offer a promising pathway to improve the production efficiency of conventional electrospinning. Collectively, this study provides a promising strategy for intelligent food packaging with potential for scale-up; however, further assessment of production scalability, long-term stability, migration behavior, and food-contact safety is required to confirm its practical applicability. The PG//PT membrane may show promise as a smart packaging material for visual freshness monitoring and preservation, with potential applications in direct food contact subject to thorough safety and migration assessments in future work.

## Figures and Tables

**Figure 1:**
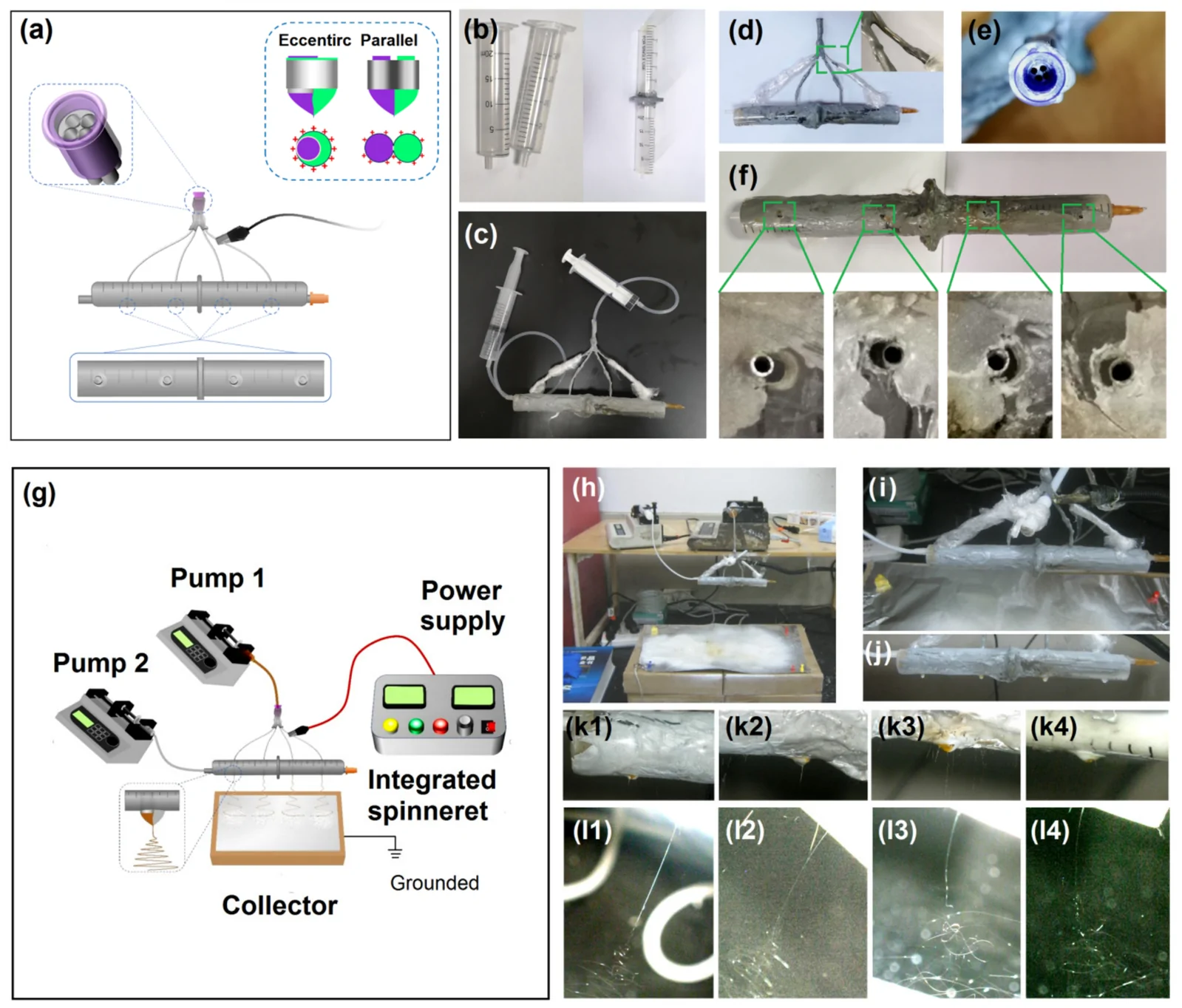
The brand-new spinneret and apparatus for batch production of PG//PT Janus nanofibers from a side-by-side electrospinning process. The homemade spinneret (**a**–**f**): (**a**) A diagram about the integrated spinneret, with its upper-right inset indicating the more reasonable arrangements of the two capillaries in the eccentric nozzle than the traditional parallel nozzle; (**b**) two 20 mL syringe shells were integrated into one liquid storage tank using epoxy resin; (**c**) a digital photo of the spinneret connected with two syringes through silicon tubes for guiding the two working fluids; (**d**) a digital image of the whole spinneret, with its upper-right inset showing a naked capillary section for introducing the electrostatic energy to the working fluids; (**e**) the inlet of the working fluids in the round needle side; and (**f**) four outlets lined up in a row and their digital images. Apparatus and working process (**g**–**l**): (**g**) A diagram about the parts in the homemade apparatus containing mainly the spinneret, two syringe pumps, a collector, and a power supply; (**h**) a photo of the working process for creating the PVDF-GSE//PVDF-TiO_2_ Janus nanofibers; (**i**) the connection details of spinneret with two working fluids and the power supply through an alligator clip; (**j**) four Taylor cones; (**k1**–**k4**) the enlarged images of the four typical Taylor cones; and (**l1**–**l4**) the typical four bending and whipping processes from the four outlets.

**Figure 2:**
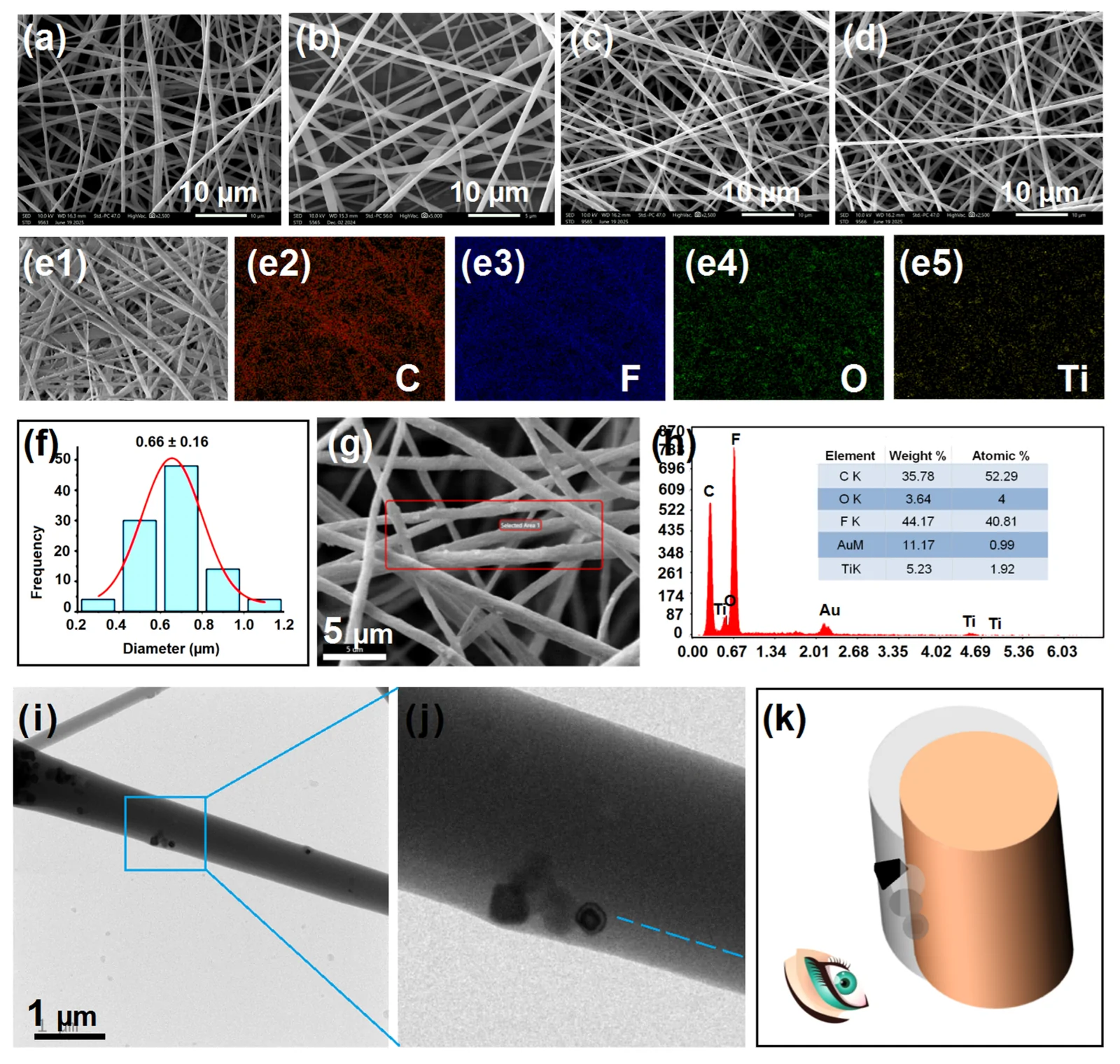
Outer morphologies and inner side-by-side structures of the resultant nanofibers. SEM images of Janus nanofibrous membranes (**a**–**d**): (**a**) P//P, (**b**) PG//P, (**c**) P//PT, and (**d**) PG//PT. Evaluation of PG//PT Janus nanofibers (**e**–**k**): (**e1**–**e5**) Element mappings of PG//PT nanofibers, C, F, O, and Ti distributions, respectively; (**f**) diameter distribution; (**g**,**h**) EDS spectrum; (**i**,**j**) TEM image; and (**k**) a diagram shows the distributions of TiO_2_ nanoparticles.

**Figure 3:**
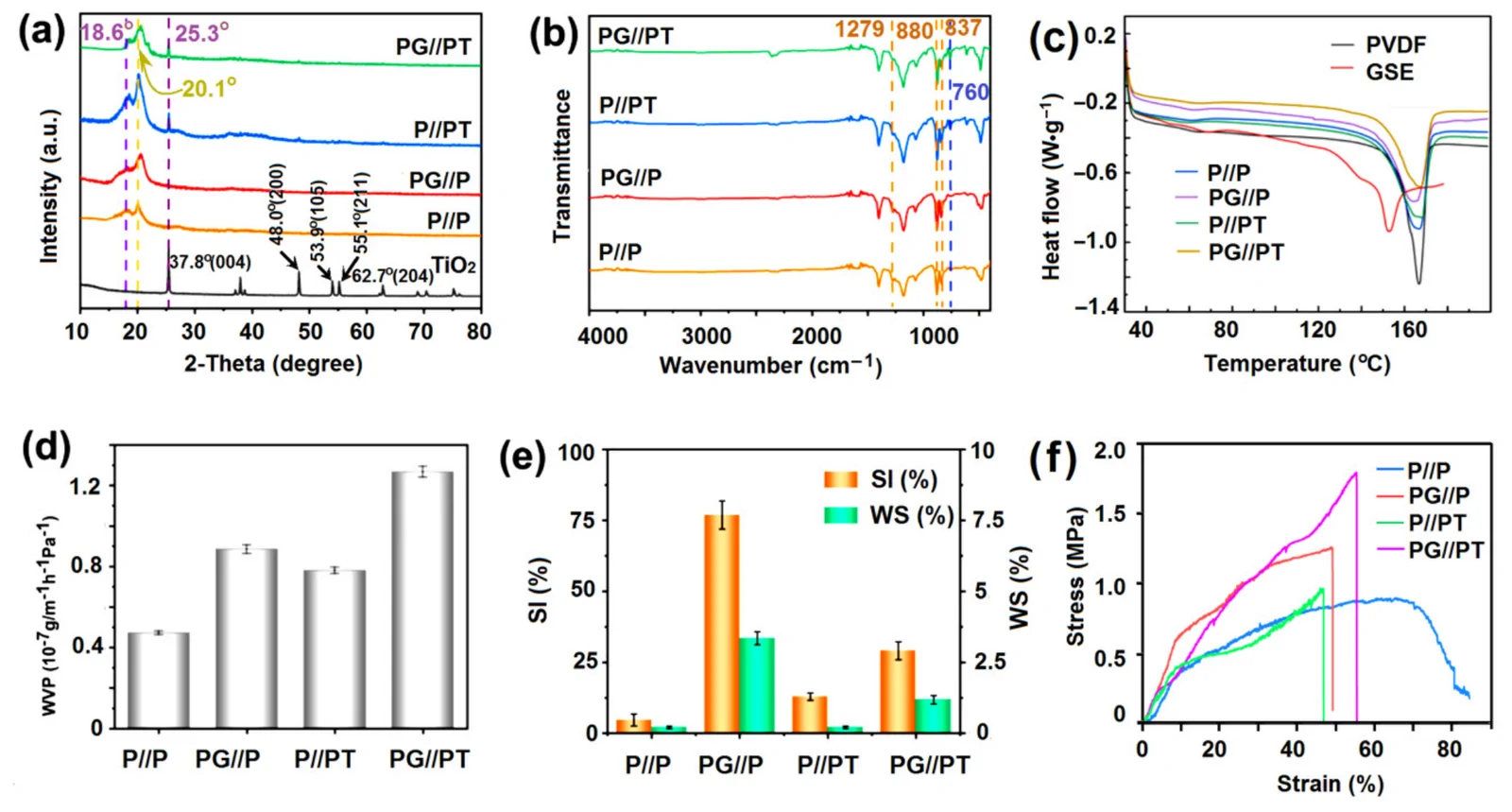
Structural and physicochemical characterization of the nanofibrous membrane: (**a**) XRD pattern; (**b**) FTIR spectrum; (**c**) DSC assessments; (**d**) water-vapor permeability; (**e**) swelling index and water solubility; and (**f**) stress–strain curve.

**Figure 4:**
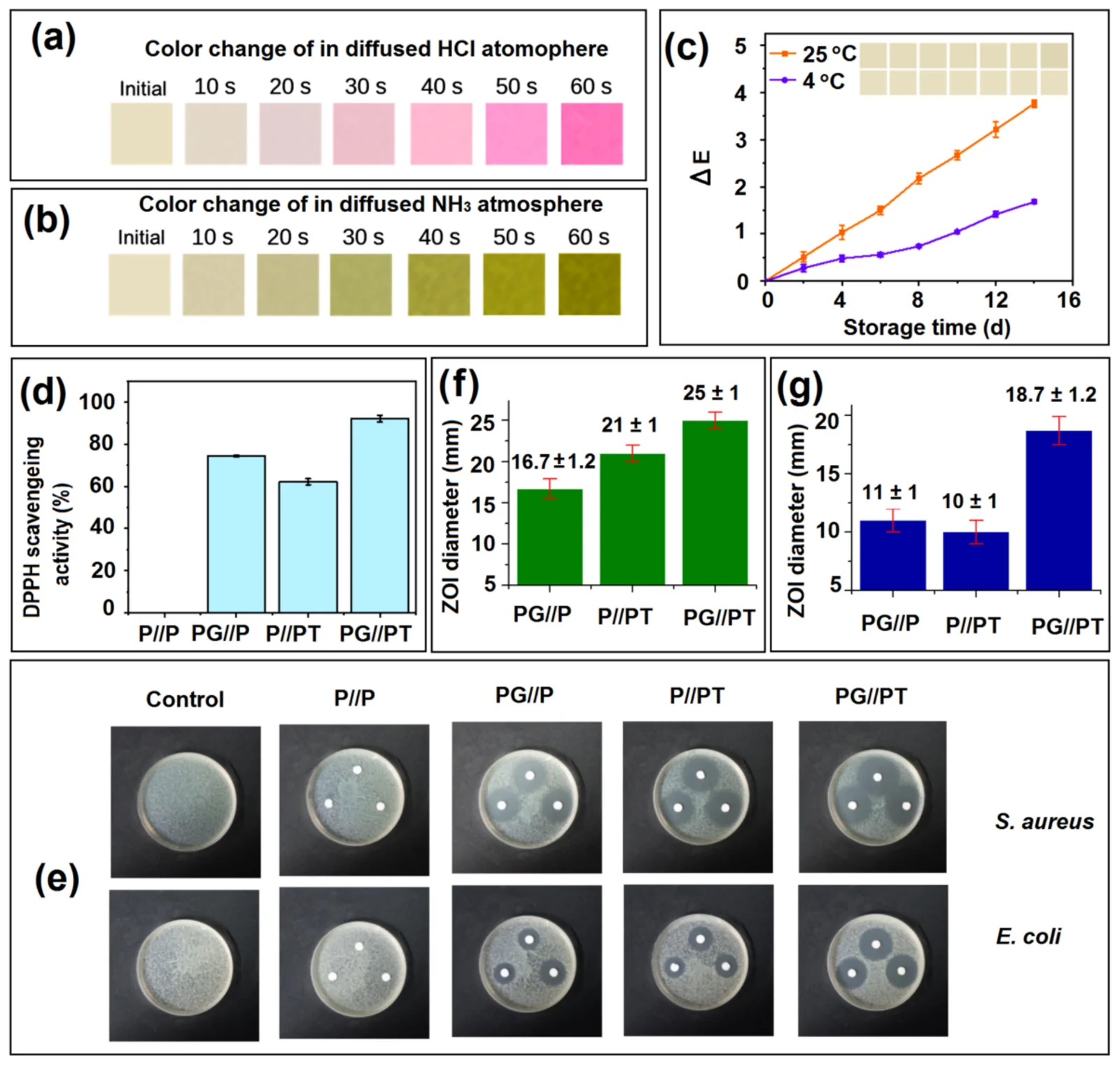
Comprehensive analysis of the color stability and freshness-preserving properties of Janus nanofibrous films. Schematic representation of color changes during the experiment, corresponding to (**a**) a concentrated hydrochloric acid environment; (**b**) a concentrated ammonia environment; (**c**) color stability of the fiber membranes after 14 days of storage at 25 °C and 4 °C; (**d**) DPPH radical scavenging activity of the membranes; (**e**) antibacterial properties of the membranes on S. aureus and *E. coli*; (**f**) the ZOI diameters of three Janus fibrous films on *S. aureus*; and (**g**) the ZOI diameters of three Janus fibrous films on *E. coli*.

**Figure 5:**
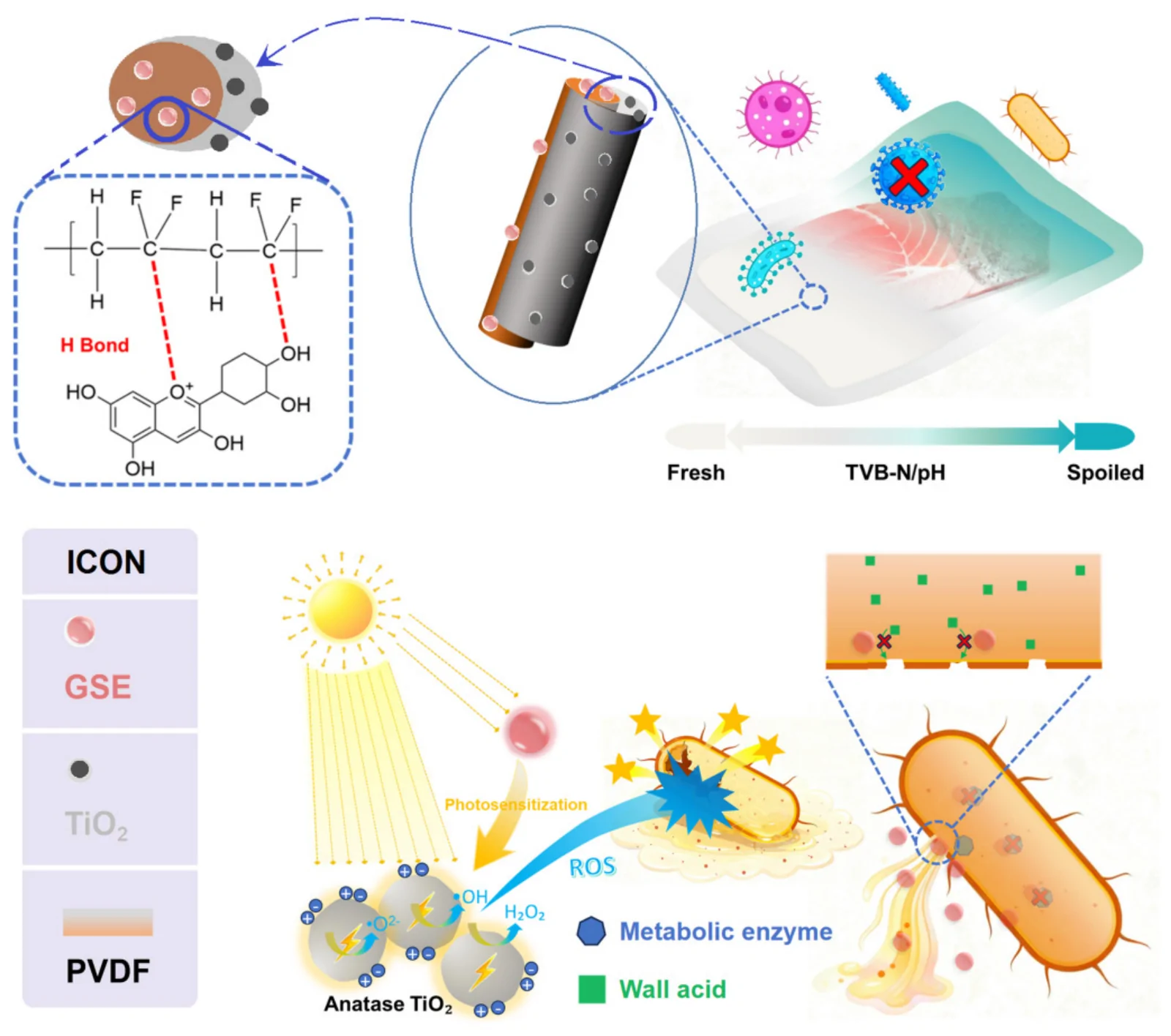
Schematic diagram of the combined antibacterial mechanism of PG//PT fiber membranes against bacteria and their intelligent monitoring and preservation functions in food packaging.

**Figure 6:**
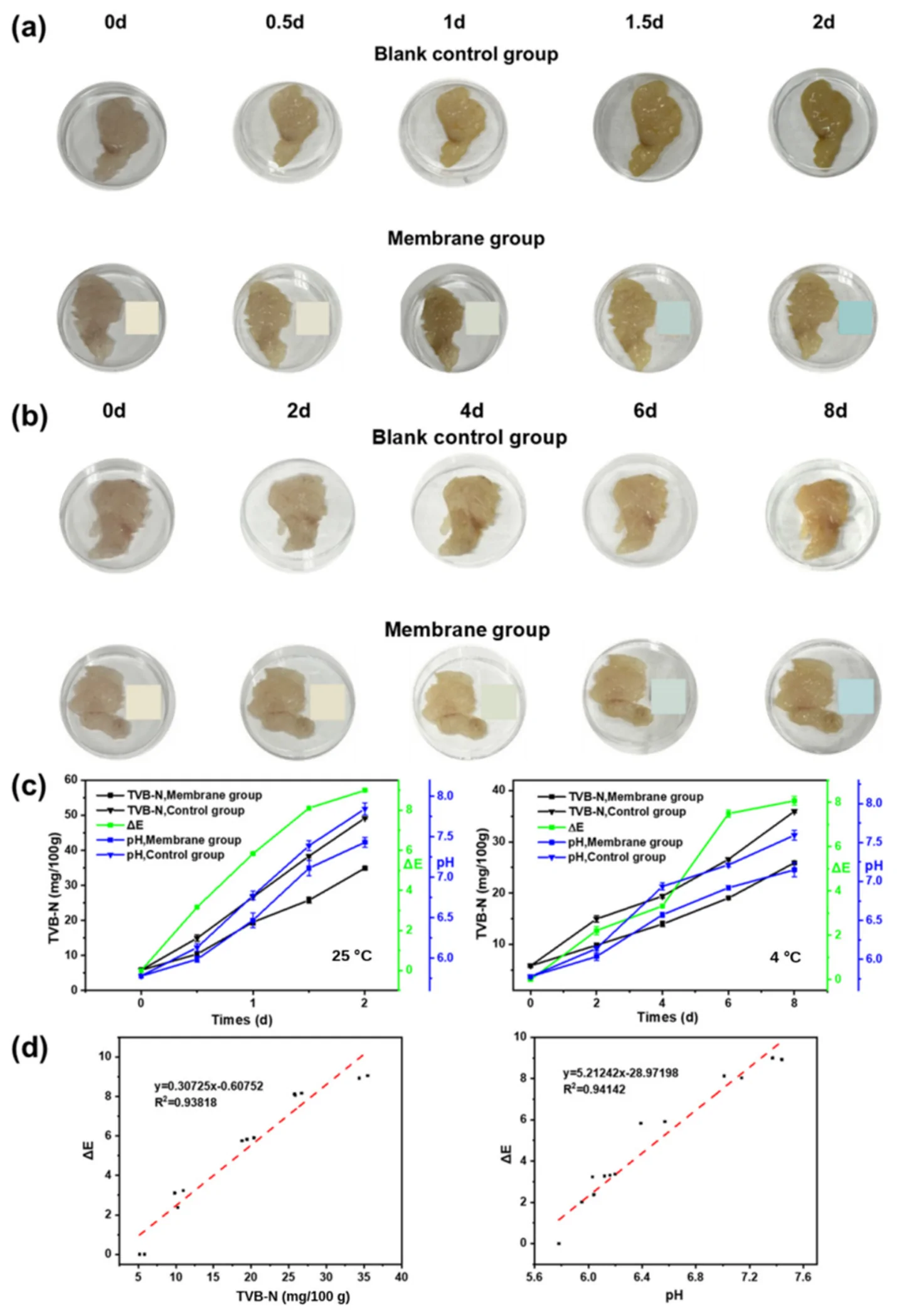
Dual-function composite fiber membrane for monitoring the freshness and preserving the quality of perch: (**a**) Stored at room temperature (25 °C); (**b**) stored in a refrigerator (4 °C); (**c**) TVB-N-, ΔE-, and pH-change curves of fresh perch during storage at 25 °C and 4 °C; and (**d**) correlation between TVB-N, pH, and ΔE of perch samples.

**Table 1:** WCA, WVP, SI, WS, TS, and EI of the membrane (mean ± SD, n = 3).

Physical Parameters	P//P	PG//P	P//PT	PG//PT
WCA (°)	134.97 ± 1.75	122.40 ± 0.98	124.30 ± 1.45	120.57 ± 0.97
WVP (10^−7^ g m^−1^ h^−1^ Pa^−1^)	0.47 ± 0.01	0.88 ± 0.02	0.78 ± 0.02	1.27 ± 0.03
SI (%)	4.67 ± 2.08	76.90 ± 5.00	12.93 ± 1.31	29.10 ± 3.15
WS (%)	0.21 ± 0.04	3.35 ± 0.23	0.21 ± 0.04	1.18 ± 0.15
σ_u_ (MPa)	0.789 ± 0.296	1.510 ± 1.217	1.030 ± 0.606	1.692 ± 0.188
ε_b_ (%)	90.14 ± 20.58	52.87 ± 34.51	51.39 ± 7.78	54.18 ± 13.59
E (MPa)	2.80 ± 2.28	4.77 ± 2.25	1.06 ± 0.28	3.96 ± 1.09
U_t_ (MJ/m^3^)	0.501 ± 0.274	0.621 ± 0.681	0.287 ± 0.194	0.524 ± 0.175

## Data Availability

The original contributions presented in this study are included in the article/Supplementary Materials. Further inquiries can be directed to the corresponding authors.

## Supplementary Materials

The following supporting information can be downloaded at https://www.mdpi.com/article/10.3390/foods15193391/s1, Figure S1. The topical deformation of syringe due to an accident firing and its possible reasons, suggesting more attention should be paid to safety; Figure S2. (a) Color changes, (b) ultraviolet-visible spectra, and (c) structural transformations of GSE solutions at different pH values; Figure S3. PVDF nanofibers with various GSE concentrations and their size distributions: (a) PVDF-G0, (b) PVDF-G5, (c) PVDF-G10, (d) PVDF-G15; Figure S4. SEM images of four types of fibers in batch spinning. SEM images of (a1–a4) P//P, (b1–b4) PG//P, (c1–c4) P//PT, and (d1–d4) PG//PT from different imaging positions; Video S1: The electrospinning apparatus and the experimental parameters.
