# Supplementary material for: Batch Productions of Electrospun Janus Nanofibrous Films for Multifunctional Food-Packaging Applications
Source: Foods. 2026 Sep 23;15(19):3391. doi: 10.3390/foods15193391 (PMC13664405; doi:10.3390/foods15193391)
Supplement: Supplementary file 1 [file foods-15-03391-s001.zip › Supplementary Materials.pdf]

Supporting information

Batch Productions of Electrospun Janus Nanofibrous Films for Multifunctional Food-Packaging Applications

Tingyu Chen, Qiuyun Guo, Hongyi Yin, Mengyao Wang, Hui Liu * and Deng-Guang Yu *

School of Materials and Chemistry, University of Shanghai for Science and Technology, 516 Jungong Road, Shanghai 200093, China;
243403094@st.usst.edu.cn (T.C.); 233393129@st.usst.edu.cn (Q.G.);
255393101@st.usst.edu.cn (H.Y.); 254203053@st.usst.edu.cn (M.W.)
* Correspondence: huiliu@usst.edu.cn (H.L.); ydg017@usst.edu.cn (D.-G.Y.)

Section 1: Developments of the new concept combined spinneret and the new side-by-side electrospinning apparatus

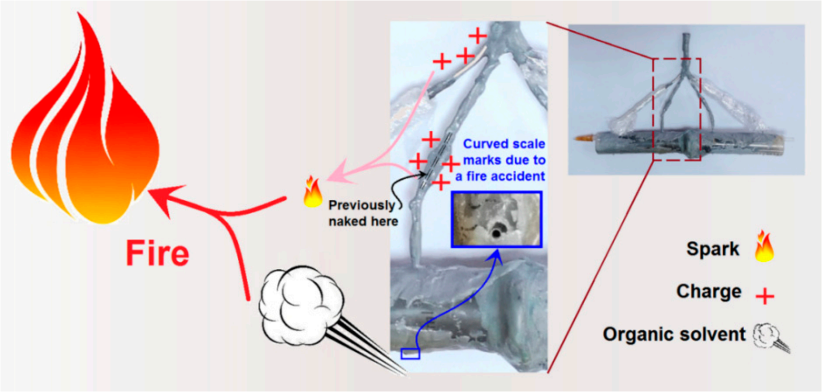

Figure S1. The topical deformation of syringe due to an accident firing and its possible reasons, suggesting more attention should be paid to safety.

Section 2: GSE pH-dependent color changes, ultraviolet-visible spectra, and structural transformations under various pH values.

The experimental results illustrated in **Figure S2** indicate that the pH-dependent color change arises from structural transformations in anthocyanins. At strongly acidic pH 3, the GSE solution adopts a red hue, attributed to the presence of flavylium cations; specifically, its maximum absorption peak occurs around 522 nm. With increasing pH, the flavylium cations undergo hydration to form colorless carbinol pseudobases. Consequently, the solution transitions to a purple hue, accompanied by a shift in the UV absorption peak to approximately 600 nm. Upon further deprotonation, the solution transitions from purple (pH = 7) to blue (pH = 8), facilitated by the formation of quinoidal bases. Under strongly alkaline conditions (pH 9–11), the solution gradually adopts a yellow hue due to increased concentrations of quinoidal bases, chalcone forms, and chalcone forms as the pH rises. The intensity of the maximum absorption peak exhibits significant pH-dependent variation, reaching its highest intensity at pH 4.0 and gradually diminishing as the pH increases. Collectively, these findings demonstrate that GSE undergoes distinguishable color changes across varying pH levels, making it a viable candidate for applications as a pH-sensitive color indicator.

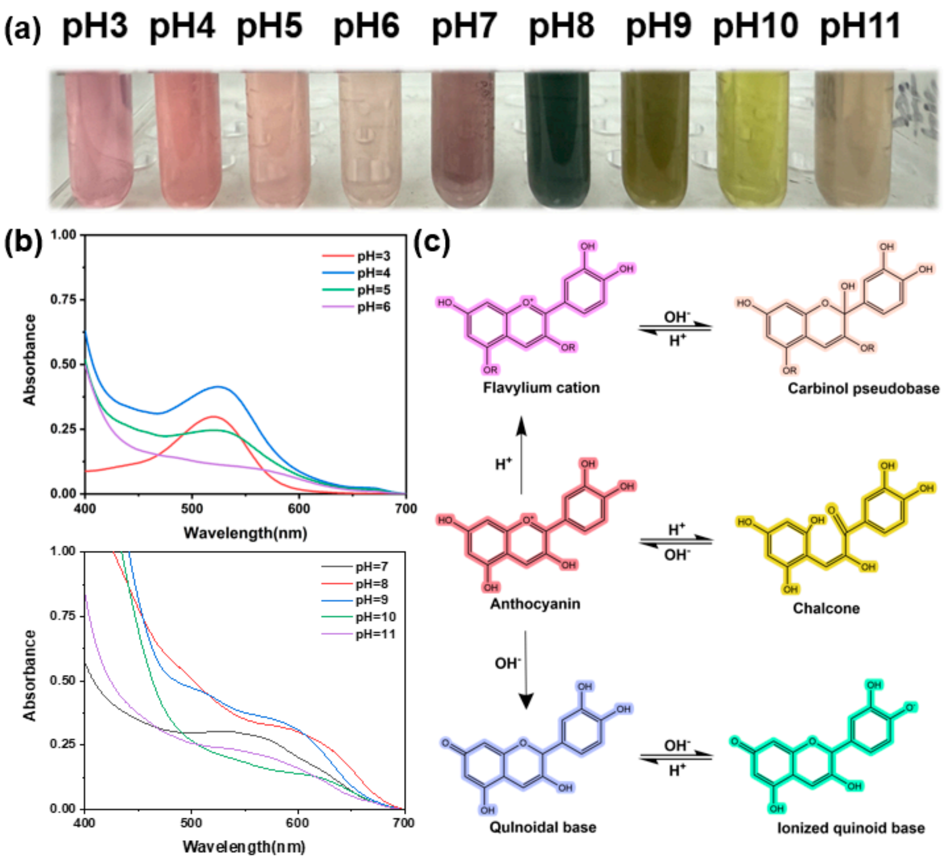

Figure S2. (a) Color changes, (b) ultraviolet-visible spectra, and (c) structural transformations of GSE solutions at different pH values.

Section 3: Needle-based electrospinning for investigating the influences of the GSE contents on the resultant PVDF-GSE nanofibers

PVDF-GSE nanofiber membranes exhibiting a monolithic structure were fabricated via the blended electrospinning method. Initially, PVDF powder was dissolved in a 3:2 (v:v) acetone/DMF solvent mixture. After thorough mixing, an 19% (*w*/*v*) PVDF solution was obtained. Subsequently, GSE powders were added at the concentrations of 5%, 10%, and 15% relative to the PVDF mass. The mixture was subjected to continuous magnetic stirring at 45 °C for a duration of 2 hours, and subsequently underwent ultrasonication for 30 minutes to eliminate bubbles, resulting in the electrospinnable working fluid.

Under an applied voltage of 11 kV, a solution feed rate of 0.8 mL h^{-1}, and a fiber collection distance of about 20 cm, the needle-based electrospinning process was

conducted. All processes were performed under the ambient conditions of a temperature of 16 ± 2 ℃ and a relative humidity of 36 ± 5%. Nanofibrous membranes with varying anthocyanin ratios were prepared and designated as PVDF-G0, PVDF-G5, PVDF-G10, and PVDF-G15.

As the anthocyanin concentration increases, the fiber morphology exhibits some variations (**Figure S3a-d**). The PVDF-G5 fibers exhibit a reduced diameter yet demonstrate uneven distribution (**Figure S3b**). In contrast, PVDF-G15 fibers are thicker and display a broader diameter distribution, accompanied by inter-fiber adhesion (**Figure S3d**). This occurs due to the increase in spinning solution viscosity at higher anthocyanin concentrations, thereby enhancing fiber adhesion during deposition onto the receiving plate. Conversely, PVDF-G10 demonstrates superior morphology characterized by uniform fiber diameter and a smooth surface texture (**Figure S3c**). At a 10% anthocyanin concentration, the polar hydroxyl groups in anthocyanin molecules form hydrogen bonds with the PVDF C–F groups, achieving an optimal balance between chain disruption and structural integrity. This interaction moderately disrupts PVDF molecular chain entanglement, yielding a plasticizing effect that lowers the glass transition temperature of the system and enhances segmental mobility. Notably, this occurs without significantly compromising the tensile strength of the material, thereby promoting uniform fiber formation. Concurrently, during rapid evaporation of the acetone/DMF solvent mixture, hydrogen bond crosslinking at the 10% anthocyanin concentration effectively immobilizes anthocyanin molecules within the PVDF fiber network, enabling submicron-level homogeneous dispersion. This synergizes with the steric hindrance imparted by the anthocyanin benzopyran rigid framework, eliminating surface roughness, preventing macrophase separation, and promoting β-phase crystallization in PVDF molecules.

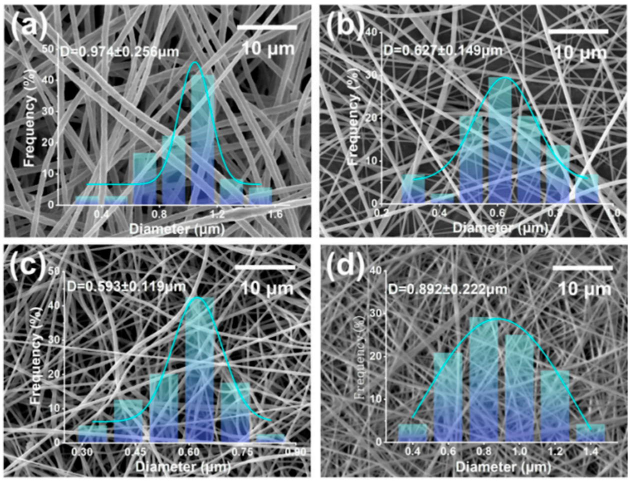

Figure S3. PVDF nanofibers with various GSE concentrations and their size distributions: (a) PVDF-G0, (b) PVDF-G5, (c) PVDF-G10, (d) PVDF-G15.

Section 4: **Uniformity of the Janus nanofibers from different outlets**

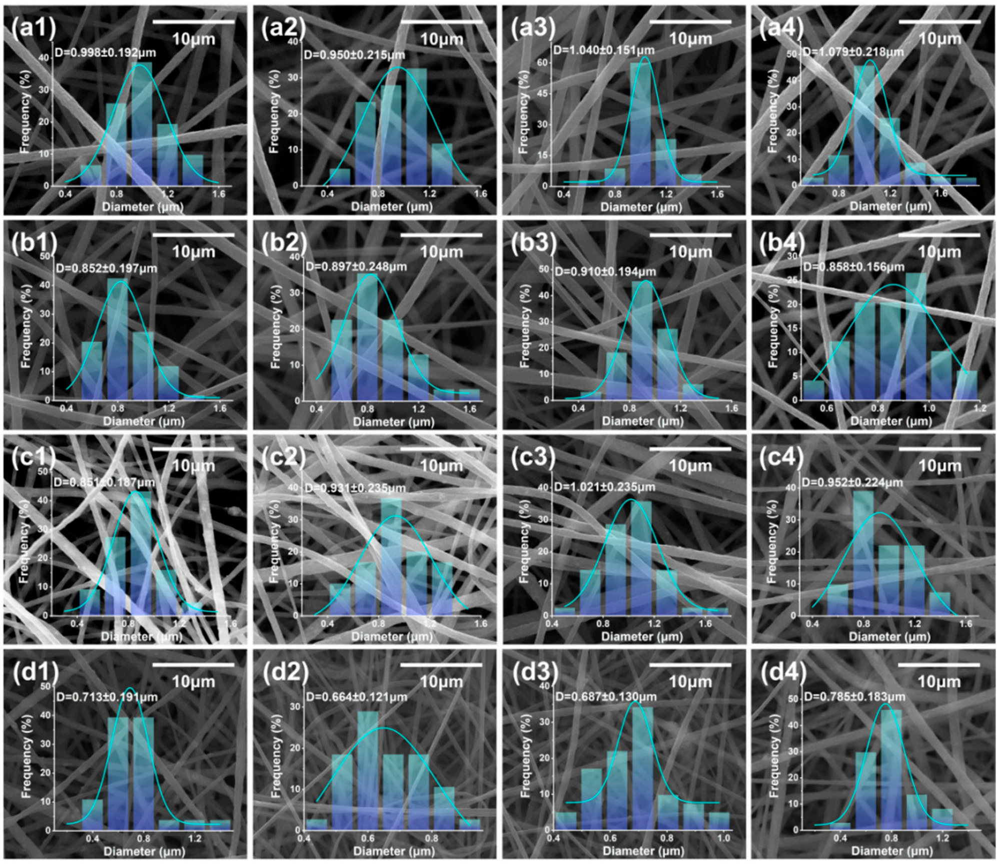

Figure S4. SEM images of four types of fibers in batch spinning. SEM images of (a1-a4) P//P, (b1-b4) PG//P, (c1-c4) P//PT, and (d1-d4) PG//PT from different imaging positions.
